# Selective pressure of endocrine therapy activates the integrated stress response through NFκB signaling in a subpopulation of ER positive breast cancer cells

**DOI:** 10.1186/s13058-022-01515-1

**Published:** 2022-03-09

**Authors:** Svetlana E. Semina, Purab Pal, Nidhi S. Kansara, Rosemary J. Huggins, Elaine T. Alarid, Geoffrey L. Greene, Jonna Frasor

**Affiliations:** 1 Department of Physiology and Biophysics, University of Illinois at Chicago, 835 S. Wolcott Ave, E202 MSB, MC901, Chicago, IL 60612 USA; 2 Ben May Department for Cancer Research, University of Chicago, Chicago, IL 60637 USA; 3 Department of Oncology, University of Wisconsin-Madison, Madison, WI 53705 USA

**Keywords:** Breast cancer, Estrogen receptor, Endocrine therapy, NFκB, Integrated stress response

## Abstract

**Background:**

While estrogen receptor (ER) positive breast tumors generally respond well to endocrine therapy (ET), up to 40% of patients will experience relapse, either while on endocrine therapy or after ET is completed. We previously demonstrated that the selective pressure of tamoxifen activates the NFκB pathway in ER + patient tumors, breast cancer cell lines, and breast cancer xenograft tumors, and that this activation allows for survival of a subpopulation of NFκB + cells that contribute to cell regrowth and tumor relapse after ET withdrawal. However, the mechanisms contributing to the expansion of an NFκB + cell population on ET are unknown.

**Methods:**

Here, we utilized single-cell RNA sequencing and bioinformatics approaches to characterize the NFκB + cell population and its clinical relevance. Follow-up studies were conducted to validate our findings and assess the function of the integrated stress response pathway in breast cancer cell lines and patient-derived models.

**Results:**

We found that the NFκB + population that arises in response to ET is a preexisting population is enriched under the selective pressure of ET. Based on the preexisting NFκB + cell population, we developed a gene signature and found that it is predictive of tumor relapse when expressed in primary ER + tumors and is retained in metastatic cell populations. Moreover, we identified that the integrated stress response (ISR), as indicated by increased phosphorylation of eIF2α, occurs in response to ET and contributes to clonogenic growth under the selective pressure of ET.

**Conclusions:**

Taken together, our findings suggest that a cell population with active NFκB and ISR signaling can survive and expand under the selective pressure of ET and that targeting this population may be a viable therapeutic strategy to improve patient outcome by eliminating cells that survive ET. Understanding the mechanisms by which breast cancer cells survive the selective pressure of ET may improve relapse rates and overall outcome for patients with ER + breast tumors.

**Supplementary Information:**

The online version contains supplementary material available at 10.1186/s13058-022-01515-1.

## Background

The majority of breast tumors express estrogen receptor α (ER) and women with ER + disease typically receive adjuvant endocrine therapy (ET), such as tamoxifen or aromatase inhibitors. While the majority of ER + tumors respond to these agents, up to 40% of tumors will eventually develop resistance and recur, often as metastatic disease [1, 2]. As a result, the majority of breast cancer-related deaths each year occur from ER + disease [3]. It is estimated that more than 50% of recurrences and 2 out of every 3 deaths from ER + breast cancer will occur after a woman has completed 5 years of adjuvant ET [4, 5]. The high frequency of late recurrences in ER + disease suggests that a population of breast cancer cells can survive ET only to regrow once therapy is completed. This idea is supported by recent clinical trials indicating that there is an added benefit of extending ET to 10 years [6]. Thus, a greater understanding of mechanisms contributing to the survival of cells on ET is urgently needed.

Numerous mechanisms contributing to ET resistance and disease relapse have been reported, including ER loss or inactivation, ER constitutive activation via ESR1 mutation, and/or activation of other signaling pathways that can compensate for altered ER function [7–15]. In particular, the NFκB pathway has been shown to be activated in ET-resistant tumors and contribute to more aggressive ER + disease through numerous mechanisms (see review [16]). While these mechanisms have largely been described in tumors and models once ET resistance has fully developed, the early responses to ET that allow for the initial survival of cells prior to development of resistance mechanisms have not been well studied. In a previous study we reported that the selective pressure of tamoxifen leads to activation of NFκB in patients treated with neoadjuvant tamoxifen, as well as in breast cancer cell lines and xenograft tumors [17]. NFκB activation was observed in a subpopulation of cells, which depended on NFκB for their survival [17]. These cells displayed a certain degree of plasticity initially but did contribute to increasing refractoriness to tamoxifen over time, suggesting these cells were tamoxifen tolerant rather than completely resistant. A study of early response to estrogen deprivation, to mimic aromatase inhibitor tolerance, also demonstrated a population-specific activation of NFκB [18]. We also showed that inhibiting NFκB prevented regrowth of cells and tumors once ET had been withdrawn [17]. These findings suggested that an NFκB + population of cells expands on ET and that these cells may contribute to eventual disease relapse.

In this study, we examined mechanisms by which an NFκB + cell population arises and survives in response to the selective pressure of multiple ETs and in multiple preclinical models of ER + disease. Using single-cell RNA sequencing, we show this population is preexisting in untreated parental MCF-7 cells, expands on ET, persists in metastatic tumors, and predicts poor outcome in human tumors. Moreover, we find that multiple cellular stress response pathways are activated in response to ET and highly correlated with NFκB activity. We further demonstrated that the Integrated Stress Response (ISR), as indicated by phosphorylation of the translational initiator, eIF2α, is activated and contributes to clonogenic growth under the selective pressure of ET. Together, these findings suggest that the rate of relapse for patients receiving ET could be improved by targeting of stress responses to eliminate cells that survive ET.

## Methods

### Reagents

4OHT (cat. # H7904) and ICI (cat. # I4409) were obtained from Sigma, and ISRIB (cat.# 16,258) was purchased from Cayman. Primary antibodies were purchased for GFP (Proteintech, cat. # 66,002–1) and phospho-eIF2α (Ser51) (Invitrogen, cat.# 701,268). Secondary antibodies, Alexa Fluor 594 (cat. # A21207) and Alexa Fluor 488 (cat.# A21202), were purchased from Invitrogen.

### Cell line and PDxO culture

The human ER + breast cancer cell lines (MCF-7 and T47D) were obtained from Dr. Debra Tonetti (University of Illinois at Chicago). MCF-7-NFκB-RE-GFP reporter cells [19] were kindly provided by Dr. Elaine T. Alarid (University of Wisconsin-Madison). T47D-NFκB-RE were generated as previously described [17]. Cell lines were cultivated in RPMI 1640 media (Gibco) with phenol red supplemented with 10% FBS, 1% non-essential amino acids, 2 mmol/L L-glutamine, and 1% antibiotics penicillin–streptomycin. All cell lines are routinely authenticated by short tandem repeat analysis with GenePrint® 10 System (Promega, cat. # B9510) and tested for mycoplasma using LookOut Mycoplasma PCR Detection Kit (Sigma). ER + patient-derived organoids (PDxOs) HCI-003 and HCI-017 were provided by Dr. Alana Welm (University of Utah, Huntsman Cancer Institute). PDxOs were cultured embedded in 100% matrigel (Corning, cat. # CB-40230) with PDxO media (Advanced DMEM, 5% FBS, 1% Glutamax, 0.1% hydrocortisone, 0.1% Gentamycin, and 0.01% hEGF) containing the following additives: 1uL/mL ROCKi, 2uL/mL NAC, 1uL/mL FGF2, and 1uL/mL estradiol (E2).

### Clonogenic assay

Clonogenic assay was conducted as previously described [17]. Briefly, cells were seeded in 6-well plate in 1,000 cells per well density in growth media (see above) and treated with 4OHT or ICI at final concentrations of 1 µM; or cells were seeded in phenol-red free media with 5% CD-FBS for estradiol deprived (ED) conditions to mimic aromatase inhibitor treatment. Media with treatment was refreshed every 3–4 days for 2 weeks. After 2 weeks, plates were scanned with a Celigo Imaging Cytometer (Nexcelom Bioscience). Confluence ratio for brightfield and GFP was calculated using the confluence application.

### PDxO growth assay

Organoid domes were washed with 1X PBS, and then 800 µl dispase (50U/ml), 200 µl FBS, and 1 µl ROCKi were combined and added to each well. Matrigel domes were scraped into the dispase mixture and resuspended to break up the Matrigel. Following incubation and washing, organoids were resuspended in Matrigel to obtain a final organoid concentration of 5,000 organoids/ml. Using a 48-well plate, 10 µl of organoids suspended in Matrigel was pipetted into each well to create a small dome. PDxOs media was supplemented 4OHT or ICI in final concentration 1 µM; or ED conditions were used (phenol-red free RPMI, 5% CD-FBS, 0.1% hydrocortisone, 0.1% Geneticin). Plates were analyzed for total organoid area per µm^2^ once every 24 h for 14 days using the Incucyte S3 organoid module to measure growth over time.

### RNA Extraction and RT-qPCR

For RNA extraction was used phenol–chloroform-based method with TRIzol (Invitrogen, cat. # 15,596,026) according to manufacturer’s protocol. RT-qPCR was performed and analyzed as described previously [16].

### Single-cell RNA sequencing (scRNA-seq)

For inDrop scRNA-seq, tamoxifen-treated cells were collected and resuspended in × 1 PBS with 0.04% BSA. Single-cell capturing and encapsulation was performed using the inDrop™ System from 1CellBio by the UIC Genome Research Core following the manufacturer’s protocol (v.2.4). After encapsulation, cells were transferred to 1CellBio UV Cleavage Device for the RT reaction. Libraries were constructed according to the inDrop Library Preparation Protocol (v2.3). Sequencing was performed using the Illumina NextSeq 500 with the High Output Kit 75 cycles. Read format was as follows: Read1 50b and wRead2 36b. Total cDNA reads output approximately 400 Mb. For 10X Genomics, untreated MCF-7 cells were collected and resuspended in × 1 PBS with 0.04% BSA. Cell suspension was loaded on a Chromium Single Cell 3′ Chip (10X Genomics). Single-cell libraries were prepared according to the manufacturer’s protocol. 10X libraries were pooled and were sequenced on an S4 lane with 28 × 150nt reads and produced over 5.4 billion reads. Data are available through Gene Expression Omnibus (GSE 181812). Raw data were processed and aligned by the UIC Research Informatics. R2 reads were mapped to the reference transcriptome (hg38 Ensemble gene sequences, exonic only) using BWA MEM. Cell barcodes and UMIs were extracted from R1 using a custom pipeline following OneCellBio adapter design for inDrop data. cellRanger (v3.0.0) was run on the raw data to align Ensembl genome GRCh38 for 10X Genomics data Unique UMI counts were summed for each gene and each unique cell barcode. Only cell barcodes with > 500 counts included in the final counts table.

Downstream analysis was performed by using the Seurat package (v.3.2) in the Rstudio (v.4.0.3). At the quality control step cells with low counts (< 2000 genes), high mitochondrial genes expression (> 10% of total mapped reads) and cell duplicates were excluded from analysis. Data from 648 single-cell transcriptomes were further scaled and normalized (NormalizeData function using normalisation.method = “LogNormalize”, scale.factor = 10,000, followed by the ScaleData function). Mitochondrial genes were regressed out to minimize their effect on clustering. Principal component analysis (PCA) was performed to measure the distance between cells. The number of principal components was determined using the JackStraw resampling method, and only the statistically significant (p < 0.05) components were used to create a KNN graph. The Uniform Manifold Approximation and Project (UMAP) reduction technique was used to visualize the data in low-dimensional space.

For the integration of data from 4OHT-treated MCF-7 cells with data from untreated MCF-7 cells [23, 24] or long-term estrogen-deprived (LTED) cells, we used publicly available datasets downloaded from Gene Expression Omnibus (GSE114462, GSE144320 and GSE122743, respectively). Integration of these datasets was performed using the SCTransform vignette in the Seurat package to reduce technical variation caused by different methods of sample processing, as recommended by Hafemeister and Satija [20].

### Functional enrichment analysis (FEA)

FEA was used to identify enrichment of gene signatures across the identified clusters [18]. Signatures tested were derived from MSigDB [21, 22] v.7.4 or custom generated from previous RNAseq data [17]. Prior to calculating signature scores, the data was normalized and scaled gene-wise. Then z-scored signature was calculated for each cell separately. ROC analysis was used to estimate the accuracy of enrichment of a signature within a particular cluster. Area Under the Curve (AUC) > 0.7 was considered an enrichment. Significance of a signature enrichment across the clusters was estimated by the Wilcoxon rank-sum test (p < 0.01 was considered significant). The Pearson’s correlation coefficient and statistical significance were calculated using Rstudio. Correlation coefficients of 0.3–0.5 indicate a moderate correlation and 0.5–0.9 indicate a strong correlation. FEA for a custom NFκB + Population Signature was performed on scRNA-seq datasets from ER + primary and metastatic patient-derived xenograft (PDX) tumors [25] (GSE131007).

### Ingenuity pathway analysis (IPA)

The IPA package (QIAGEN Redwood City, www.qiagen.com/ingenuity) was used to identify a network connecting DEGs from the NFκB + cell population (i.e., Cluster 4). The network and the type of connection between DEGs were formed based on the Ingenuity Knowledge Base repository (inferred from the scientific literature) [26].

### Co-immunofluorescence

Cells were seeded on glass coverslips and treated with ET for 2 weeks in clonogenic conditions. Cells were then fixed with 4% paraformaldehyde (PFA), permeabilized using 0.2% Triton X-100, blocked with casein, and incubated with primary antibody (1:100 dilution for anti-peIF2a and 1:800 for anti-GFP tag in casein) for 1 h at room temperature. After washing, cells were incubated with a secondary antibody (1:1000 for Alexa fluor 594 and 488 in casein) for 1 h at room temperature. Glass coverslips were washed with 1X-TBS and mounted onto glass slides using ProLong™ Gold Antifade Mountant with DAPI (Life Technologies, cat. # P36935). Images were acquired using a Leica DMi8 microscope at 63 × magnification using the same acquisition settings across all samples. Image analyses were performed by using ImageJ software. peIF2α (red) and GFP (green) fluorescence intensities were calculated for each nucleus as individual region of interests (ROI) across the image fields by Analyze Particles Function. Statistical analyses for Pearson’s correlation test and significance of ImageJ data were performed using GraphPad v.9.0.

## Results

### NFκB activation in response to the selective pressure of endocrine therapy is restricted to a specific cell population

**Fig. 1:**
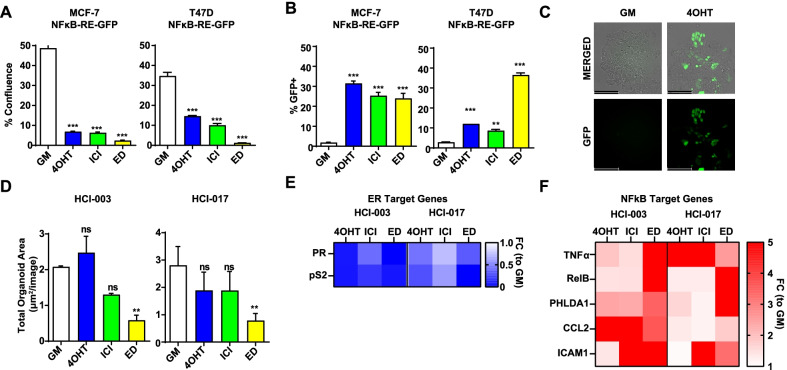
Activation of NFκB by different ETs in preclinical ER + breast cancer models. **a**–**c** Clonogenic assays were conducted in MCF-7-NFκB-RE-GFP and T47D-NFκB-RE-GFP cell lines cultured in growth media (GM) in the presence or absence of 1 μM 4OHT or ICI, or in ED conditions for 2 weeks. **a** Colony confluence (area covered by colonies) was quantified using a Celigo imaging cytometer. **b** The percentage of GFP + confluence per condition was determined using Celigo imaging. **c** Representative images of colonies for the MCF-7-NFκB-RE-GFP cell line grown in GM ± 4OHT for 2 weeks. Scale bar: 200 µm. **d** HCI-003 and HCI-017 PDxOs were grown under the selective pressure of ETs for 2 weeks. Data represent total organoid area per µm^2^ on day 14 as determined by the Incucyte S3 organoid module. **e, f** Expression of ER target genes **e** and NFκB target genes **f** was determined by QPCR in HCI-003 and HCI-017 PDxOs treated with ET for 2 weeks. The ER target genes were used as controls for ET. The heatmap represents Fold Change for each gene relative to GM control

Our previous work indicated an NFκB + cell population arises under the selective pressure of 4-hydroxytamoxifen (4OHT) in vitro, in vivo, and in tumors of patients treated with neoadjuvant TAM. However, it is unknown whether NFκB activation occurs in response to other ETs. To test this, we used NFκB-RE-GFP reporter MCF-7 and T47D cell lines, which we have previously shown respond to the selective pressure of 4OHT with an expansion of GFP + cells [17]. We cultivated these cell lines for two weeks in the presence of 4OHT or ICI 182,780 (ICI) or in estrogen-depleted conditions (ED), to mimic aromatase inhibitor treatment. As expected, we found that all treatments were growth suppressive (Fig. 1a). However, the proportion of GFP + cells was higher with ETs compared to growth media (GM) alone (Fig. 1b, c), indicative of NFκB activation in response to the selective pressure of different endocrine agents. To extend our findings into additional preclinical breast cancer models, we examined NFκB activation by ETs in two ER + patient-derived xenograft organoid (PDxO) models. While there was a trend toward reduced organoid area by ETs (Fig. 1d), a strong repression of ER target genes was noted (pS2, PR, Fig. 1e). However, both PDxOs showed an induction of known NFκB target genes (CCL2, TNF, PHLDA1, RelB, and ICAM1) to varying degrees by different ETs (Fig. 1f), suggesting that NFκB activation is a common yet heterogeneous response to the selective pressure of different endocrine agents in multiple preclinical models of ER + disease.

**Fig. 2:**
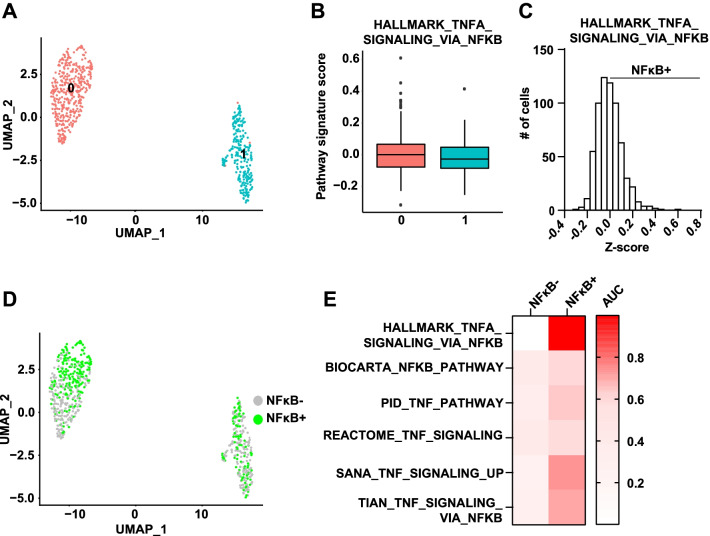
Single-cell RNA sequencing identifies an NFκB + cell population. **a** scRNA sequencing was conducted on MCF-7 cells treated with 4OHT for 2 weeks in clonogenic conditions. Bi-dimensional representation of 648 single-cell transcriptomes is shown (UMAP). **b** Box plots represent the Hallmark NFκB signature pathway score (median, interquartile values, range, and outliers) for each cell cluster. FEA for each cluster was performed but threshold for enrichment was not reached for either (Cluster 0: AUC 0.56, *P* = 0.02; Cluster 1: AUC 0.44, *P* = 0.02). **c** Bar plot showing the distribution of z-scores for the Hallmark NFκB signature on a per cell basis. **d** NFκB + cells (green) and NFκB- cells (gray), based on individual cell z-scores, are distributed across cell clusters defined in **a**. **e** FEA was performed for additional known NFκB genes signatures from MsigDB. AUC values are shown in a heatmap, and P-values are presented in Additional file 2: Table S1

Interestingly, we observed that not all cells treated with ETs were NFκB + (Fig. 1c), indicating that NFκB activation is heterogeneous and may be restricted to a specific subpopulation of cells. To examine this NFκB + cell population in more detail, we performed scRNA-seq of MCF-7 cells treated with 4OHT for 2 weeks. Unsupervised clustering revealed two distinct clusters of cells (Fig. 2a). However, neither cluster was enriched for expression of a hallmark NFκB signature [21] (Fig. 2b), suggesting that NFκB is not a driver of cell clustering. To identify NFκB + cells, we performed z-scoring of individual cells for expression of the hallmark NFκB signature and found that ~ 40% of all cells were positive (Fig. 2c), as indicated by a z-score above 0, and that these cells were equally distributed across the two clusters (Fig. 2d). To confirm that we have identified the NFκB + cell population, we performed functional enrichment analysis (FEA) for additional known NFκB signatures and found that multiple signatures were also enriched in the NFκB + cells identified by the z-scoring method (Fig. 2E, Additional file 2: Table S1). Thus, these findings confirm that NFκB activation is restricted to a specific subpopulation of cells in response to the selective pressure of ET.

### *The NFκB* + *cell population is preexisting, enriched by the selective pressure of ET, persists in ET resistance, and is predictive of aggressive tumors and disease relapse*

**Fig. 3:**
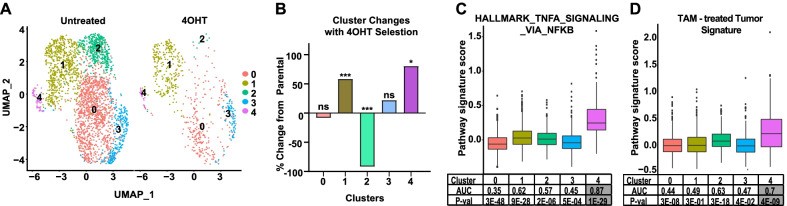
Integration of two single-cell RNA sequencing datasets from untreated and 4OHT-treated MCF-7 cells. **a** Single-cell transcriptomes from 2618 parental MCF-7 cells and 648 4OHT-treated MCF-7 cells were integrated and represented bi-dimensionally using Seurat package v.3.1. **b** The percent change in abundance of cell populations with 4OHT treatment relative to the total population for each group are shown. *P < 0.05, ***P < 0.001, ns = not significant. **c** FEA was performed for NFκB gene signatures with one representative example shown in box plots. AUC and P-values for other NFκB signatures are presented in Additional file 3: Table S2. **d** A custom gene signature was derived from differentially expressed genes in tumors of patients receiving neoadjuvant tamoxifen. The signature was used for FEA and the signature score per cluster is presented in boxplots

We next asked whether the NFκB + cell population arises de novo with the selective pressure of 4OHT or is a preexisting population. To address this question, we integrated the 4OHT-treated scRNA-seq dataset with a dataset from untreated parental MCF-7 cells [23]. We identified five different populations with unique transcriptional profiles (Fig. 3A), two of which were enriched with 4OHT treatment, two not different, and one depleted with 4OHT treatment compared to parental cells (Fig. 3b). FEA for NFκB activity was performed and only Cluster 4 was found to be highly enriched for well-established NFκB signatures (Fig. 3c and Additional file 3: Table S2). To further verify that Cluster 4 is the NFκB + cell population of interest, we generated a custom signature derived from ER + tumors of patients that underwent neoadjuvant TAM treatment, which we previously showed was enriched for NFκB activity [17]. We found that only Cluster 4 was enriched for the TAM-treated tumor signature (Fig. 3d). These findings indicate that Cluster 4 is a clinically relevant NFκB + cell population that is preexisting and can expand under the selective pressure of ETs. Because these findings were obtained using publicly available data from untreated MCF-7 cells, we conducted similar analysis on two other datasets of untreated MCF-7 cells, one from our laboratory and one from Dr. Oesterreich’s laboratory (Additional file 1: Figure S1 A-H, Additional file 7: Table S6). This analysis confirmed that an NFκB + cell population preexists in MCF-7 cell lines obtained from multiple sources.

We were also interested in whether the NFκB + cell population found with short term 4OHT treatment persists in long-term resistance. To address this, we took advantage of a scRNA-seq dataset from Hong et al. that was obtained from long term estrogen deprived (LTED) MCF-7 cells to mimic aromatase inhibitor resistance [18]. Unsupervised clustering of integrated datasets revealed 4 different clusters (Additional file 1: Fig S1 I, J). FEA for NFκB activity and a custom signature derived from ER + tumors of patients that underwent neoadjuvant TAM treatment showed enrichment in Cluster 2 (Additional file 1: Fig. S1K-L, Additional file 8: Table S7). Interestingly, this population was not statistically different between either LTED or 4OHT treatment, suggesting that the NFκB + cell population can persist in a long-term resistance model.

**Fig. 4:**
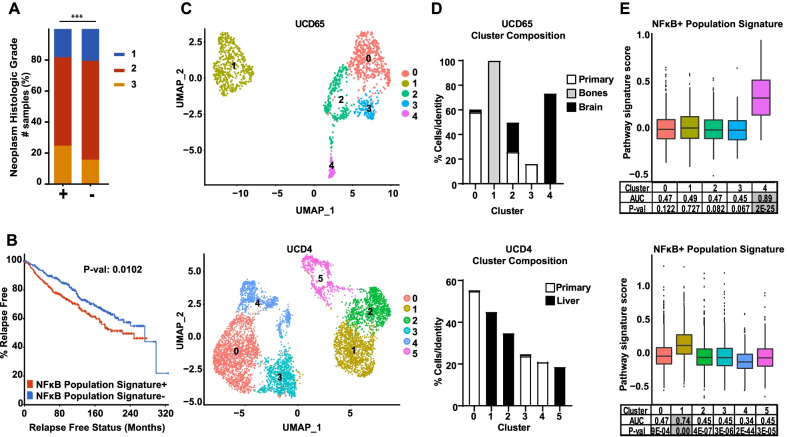
Clinical relevance of an NFκB + population signature. **a, b** The NFκB + Population Signature was interrogated in 1817 ER + breast tumors from the Metabric cohort available in cBioPortal for Cancer Genomics. Histologic grade **(a)** and patient relapse free survival **(b)** between tumors + vs. – for expression of the NFκB + Population Signature are displayed. Statistical significance was determined using chi-squared test **(a)** or log-rank test **(b)**. ***P < 0.001. **c** Single-cell transcriptomes of from primary and metastatic tumors of PDX models UCD65 (top) and UCD4 (bottom) are represented in UMAP plots. **d** The proportion of cells in each cluster is indicated by their origin (i.e., primary tumor or metastatic location). **e** FEA was performed for the NFκB + Population Signature on both datasets with box plots showing signature scores per cluster

Since the NFκB + cell population that expands with 4OHT treatment appears to be preexisting in cell lines, we next investigated whether this population might be detected in untreated human tumors, and if so, whether it is predictive of patient outcome. For this purpose, we created a custom NFκB + cell population signature (called “NFκB + Population Signature”) derived from differentially expressed genes (DEGs) from Cluster 4 (Additional file 4: Table S3) to interrogate ER + tumors from a publicly available database [27, 28]. It was found that tumors expressing this signature were more likely to be high grade (Fig. 4a) and associated with an increased risk of relapse (Fig. 4b). We also asked whether the NFκB + cell population may be involved in tumor metastasis. To address this question, we utilized publicly available scRNA-seq datasets from ER + primary and metastatic patient-derived xenograft (PDX) tumors, UCD65 and UCD4 [25]. For the UCD65 PDX model, we identified 5 cell clusters and for UCD4 we identified 6 cell clusters (Fig. 4c), with each cluster consisting of a different ratio of primary or metastatic cells (Fig. 4d). FEA for the NFκB + Population Signature showed enrichment in Cluster 4 of UCD65, which consists of cells from brain metastases, and Cluster 1 of UCD4, which consists of liver metastases (Fig. 4e), indicating that cell populations found in metastases retain the NFκB + Population Signature. Taken together, our findings suggest that an NFκB + cell population can be found in untreated primary ER + breast tumors prior to ET, as well as in metastatic ER + PDXs, and that the preexistence of these cells is predictive of disease relapse.

### The integrated stress response (ISR) is activated under the selective pressure of ET

**Fig. 5:**
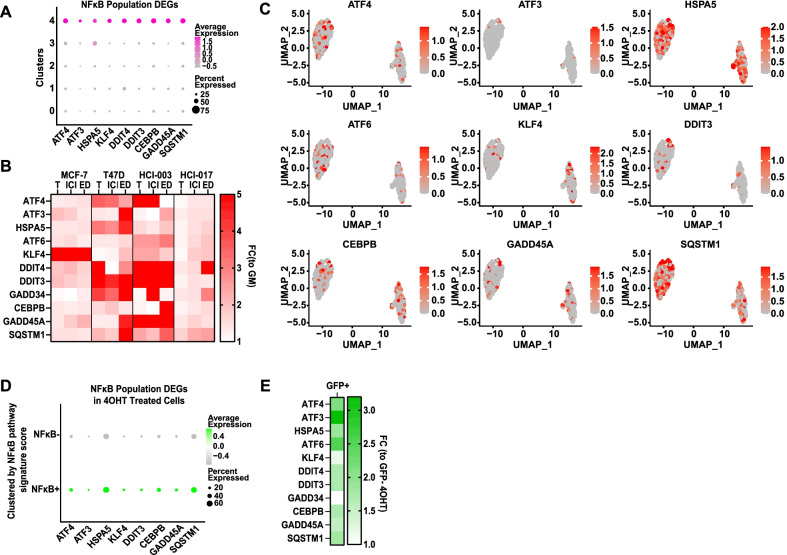
Expression of DEGs from the NFκB + cell population in multiple ER + breast cancer models treated with ETs. **a** Top DEGs in the NFκB + cell population (Cluster4) are represented in dot plots, with color representing expression level and size representing the percentage of cells in the cluster expressing the gene. **b** Expression of DEGs was determined by QPCR in MCF-7 and T47D cell lines and HCI-003 and HCI-017 PDxOs treated with different ETs for two weeks. The heatmap represents Fold Change for each gene relative to GM control. **c** Expression of top DEGs in the original 4OHT-treated cell populations from Fig. 2a are displayed on UMAP plots, representing gene expression level. **d** Expression of top DEGs is represented in dot plots for NFκB + vs NFκB- cell populations based on NFκB pathway signature score, as in Fig. 2. **e** Expression of DEGs was determined by QPCR for sorted GFP + cells relative to GFP- cells from the MCF-7-NFκB-RE-GFP cell line treated with 4OHT for 2 weeks

To understand which genes and pathways are active in the NFκB + cell population, we defined the top DEGs relative to other populations (Fig. 5a, Additional file 4: Table S3). We first examined expression of these DEGs in multiple ER + models following two weeks under the selective pressure of 4OHT, ICI, or ED. We found that most of the DEGs from the NFκB + cell population were up-regulated to varying degrees by each ET agent in each model (Fig. 5b). Importantly, some agent- and model-specific differences were observed, which may reflect either i) different regulatory mechanisms specific to a particular model or agent, or ii) the limitation of assessing cell population specific DEGs within an entire bulk population of treated cells. Thus, to address the cell population specificity of DEGs, we first used a bioinformatics approach and found heterogeneous expression of DEGs across the original 4OHT-treated cell clusters (Fig. 5c). Next, to understand whether this expression pattern was related to NFκB activity, we clustered 4OHT-treated cells into NFκB + and NFκB- populations based on their NFκB pathway signature score, as demonstrated in Fig. 2c and d. We found that the average expression of top DEGs is higher in NFκB + compared to NFκB- cells (Fig. 5d). And finally, we examined expression of the DEGs in the NFκB-RE-GFP reporter cells. We isolated GFP + and GFP- cells from the MCF-7-NFκB-RE-GFP reporter cell line after two weeks of 4OHT treatment and found that the majority of genes specific for the NFκB + cell population were, in fact, more highly expressed in GFP + compared to GFP- cells (Fig. 5e). Together these findings confirm expression of top DEGs specifically in the NFκB + cell population.

**Fig. 6:**
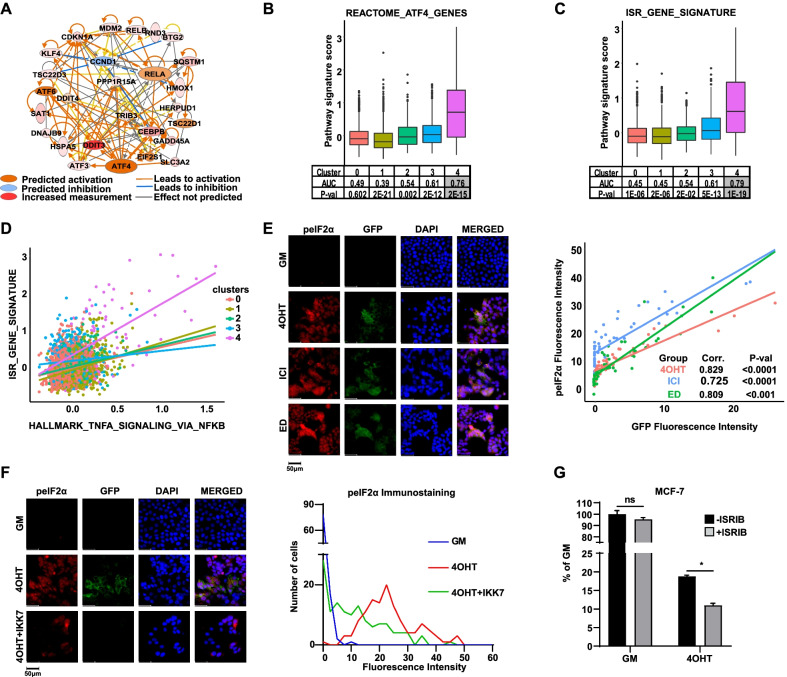
ISR pathway activation in the NFκB + cell population under selective pressure of ETs. **a** IPA network analysis for DEGs of the NFκB + cell population. Two central nodes were identified, p65/RelA (activation z-score = 2.017, *P* = 1.42E-05) and ATF4 (activation z score = 3.549, *P* = 1.04E-15). **b, c** FEA was performed to examine enrichment of ATF4 and ISR gene signatures across clusters. **d** Correlation between a hallmark NFκB signature and the ISR gene was examined for each cluster. Correlation was calculated using Pearson’s Correlation function in RStudio software (r = 0.56; *P* = 0.001). **e** Co-immunofluorescence for peIF2α and GFP was performed on MCF-7-NFκB-RE-GFP cells treated with different ETs for 2 weeks in clonogenic conditions. Scatter plot shows analysis of fluorescence intensity for peIF2α and GFP per cell in each treatment group. Intensity was measured by ImageJ software and Pearson’s correlation coefficients and P-values are shown on the graph. Scale bar: 50 µm. **f** Co-immunofluorescence for peIF2α and GFP was performed as described above. IKK7 (1 µM), an inhibitor of IKKα/β, was added for the last 72 h of culture. Histograms show fluorescence intensity of peIF2α vs. number of cells analyzed in each treatment groups. Scale bar: 50 µm. **g** The effect of an ISR inhibitor (ISRIB, 10 µM) was examined on growth of GM and 4OHT-treated cells after 2 weeks of treatment. *P < 0.05, ns = not significant

While several of the DEGs found in the NFκB + cell population (e.g., ATF3, KLF4, CEBPB, SQSTM1) are included in well-established NFκB signatures [21, 29–32], others are not (e.g., SLC3A2, EIF4A2, TERF2IP, SNHG12). Thus, to understand how these DEGs might be related to each other and to NFκB, we performed Ingenuity Pathway Analysis (IPA) and found a united network was formed with two central nodes, the NFκB family member RelA/p65 and ATF4 (Fig. 6a). Moreover, an independent ATF4 gene signature is found to be highly enriched in the NFκB + cell population (Fig. 6b). It is well established that ATF4 is a key player in ISR, a complex cellular response to sublethal stress. Accordingly, we showed that an ISR gene signature is also enriched in the NFκB + cell population (Fig. 6c) and highly correlated with NFκB activity, specifically in the NFκB + Cluster 4 cell population (Fig. 6d). It is known that ISR is activated in response to various stressors, and accordingly, we find that the NFκB + cell population is enriched for multiple stress and survival pathways, including P53, unfolded protein response (UPR), apoptosis, hypoxia, and UV response (Additional file 5: Table S4), and that these signatures are highly correlated with the NFκB signature specifically in Cluster 4 (Additional file 6: Table S5).

In response to these stressors, ISR kinases (i.e., GCN2, PKR, PERK, HRI) become activated and can phosphorylate eIF2α to reprogram protein translation to combat the stress condition. Therefore, we next asked whether ET activates ISR by examining phosphorylation of eIF2α (peIF2α) and whether this activation is specific for NFκB + cells. Interestingly, we found that the selective pressure of 4OHT, ICI or ED leads to a substantial increase of peIF2α compared to GM and that this activation is highly correlated with co-activation of NFκB based on GFP activity (Fig. 6e). To determine whether NFκB may regulate ISR, we used the NFκB pathway inhibitor IKK7 for the last 72 h of the 2-week 4OHT treatment and found a reduction in peIF2α levels (Fig. 6f), suggesting that NFκB contributes to ISR activation by ET. To determine what function ISR may be playing in response to ET, we used a specific inhibitor of ISR, ISRIB, which blocks peIF2α function [33, 34], and found that ISRIB was more effective at suppressing growth of 4OHT-treated cells compared to GM (Fig. 6g), suggesting ISR may be acting downstream of NFκB to play a protective role in response to ET.

## Discussion

This study identifies an NFκB + subpopulation of ER + breast cancer cells that are enriched in response to ET and highlights activation of stress response pathways as an underlying mechanism for the expansion of this cell population. While there is a long history linking NFκB and cellular stress to ET resistance, particularly by the Clarke laboratory [35–38], the work reported here highlights two unique aspects of this connection that have yet to be appreciated. First, the studies we conducted are looking at early adaptive responses to multiple ET agents in ET-sensitive preclinical models of breast cancer. And second, our studies highlight the heterogenous nature of NFκB activity in ER + disease by defining a unique NFκB + cell population. Importantly, the NFκB + cell population appears to be preexisting in MCF-7 cells, persists in resistance, and expression of a signature derived from this population in primary tumors prior to ET is predictive of higher-grade disease and reduced relapse free survival. Moreover, a cell population expressing the NFκB + Population Signature was detected in metastatic tumors of PDXs that were derived from breast cancer patients heavily treated with ET and/or chemotherapy. These findings suggest that the presence of an NFκB + population in ER + tumors prior to ET allows for a greater number of cells to survive ET and contribute to the eventual development of ET-resistant and metastatic disease.

Our previous work indicated that NFκB activation following ET treatment was restricted to a population of cells that retained ER but proliferated, albeit slowly, despite the presence of 4OHT [17]. Use of scRNA-seq and various bioinformatics approaches has allowed us to define this population in detail and identify potential mechanisms underlying their survival. Here, we show that this population demonstrates a unified stress-response mechanism, with NFκB and ATF4 as central regulators, that enables these cells to grow and/or survive under the selective pressure of different ETs. Bioinformatics analysis further suggested ISR as a key pathway activated in NFκB + cells, which we confirmed by demonstrating increased phosphorylation of eIF2α with ETs. ISR is considered to be a protective cellular response to sublethal stress, such as amino acid deprivation, hypoxia, unfolded protein response, or viral infection. In response to these stressors, stress kinases (i.e., GCN2, PKR, PERK, HRI) become activated and phosphorylate eIF2α, resulting in a cellular reprogramming of protein translation. While synthesis of most proteins is reduced, selective activation of other pathways occurs. In particular, the selective activation of ATF4 and ATF4 targets occurs in response to ISR so that cells can respond to stress through mechanisms that limit cellular damage (i.e., up-regulation of autophagy and anti-apoptotic mechanisms). However, if cellular stress becomes overwhelming, cell death programs can be turned on instead. Our studies suggest that the selective pressure of ET creates a sublethal stress condition to activate ISR, primarily in the NFκB + cell population. Moreover, we suggest that ISR activation allows for growth and/or survival of cells on ET, as demonstrated by the use of ISRIB, a small-molecule ISR inhibitor that can rescue translation when eIF2α is phosphorylated under chronic but not toxic conditions [33, 39].

Crosstalk between NFκB and ATF4 has yet to be studied in ER + breast cancer, and likewise, the mechanistic relationship between NFκB and ISR remains to be clarified. One previous study indicated that synthesis of IκB proteins, which inhibit NFκB activity, are selectively reduced by ISR, thereby leading to an increase in NFκB activity [40]. However, our studies suggest that NFκB may in fact be upstream of ISR, as inhibition of IKKα/β substantially reduced ISR activation. While the mechanism by which NFκB contributes to ISR is unknown, some ATF4 targets and key players in the ISR pathway, such as HSPA5 [41–44], SQSTM1 [45], and the unfolded protein response (UPR) [46, 47] pathway in general, are known to contribute to ET resistance, emphasizing the potential importance of this population to the development of resistance. Interestingly, we find that all ET agents used produced similar, although not identical, responses in the short term. Others have suggested that the selective pressure of therapy allows for survival of populations that allow for the eventual development of resistant populations through varying mechanisms [48]. Hence, we suggest that the expansion of an NFκB + cell population in response to ET could represent a precursor to the development of the various resistance mechanisms associated with ETs.

One finding of note was the varying responses of different models to different ETs, not only at the transcriptomic level but also at the cellular level. For example, both PDxOs responded very well to ED in terms of organoid size but less so to 4OHT or ICI, whereas HCI-003 PDxO responded equally well to ETs in terms of ER target gene regulation. NFκB activation and expression of NFκB + cell population DEGs in response to different ETs also varied between the cell lines and PDxOs. There are numerous explanations for these therapy and model-specific differences, including inter- and intratumoral heterogeneity and different genetic and epigenetic backgrounds of patients. It is well established that epigenetic changes, such as DNA hypo/hyper-methylation, histone acetylation/deacetylation, and micro-RNA-based alterations, can contribute to breast cancer heterogeneity [49]. Additional model-specific factors, varying degree of estrogen dependence and differences in ETs’ mechanism of actions may also account for the heterogeneity observed.

We have previously demonstrated that targeting NFκB in combination with 4OHT in ET-sensitive breast cancer models, prior to the development of resistance, can prevent cell regrowth and tumor recurrence after withdrawal of ET [17]. However, NFκB inhibition is unlikely to be a clinically viable option for cancer patients given NFκB’s broad physiological role, particularly in the immune system [50–52]. Therefore, other mechanisms, such as ISR, which contribute to the survival of NFκB + cells offer additional targets for the development of novel therapeutic strategies.

## Conclusions

Taken together, the work presented here highlights the identification of a clinically relevant NFκB + breast cancer cell population that is preexisting, enriched on ET, and predictive of increased risk of disease relapse. Activation of NFκB is highly correlated with activation of multiple cellular stress pathways, including the ISR, suggesting that the selective pressure of ET induces a sublethal stress, which NFκB-regulated ISR can protect against. We suggest that targeting ET-induced cellular stress in combination with ET may limit the survival of breast cancer cells on ET, prevent relapse, and improve overall outcomes for ER + breast cancer patients.

## Supplementary Information

**Additional file 1:** Supplemental Figure 1 showing integration of scRNA-seq datasets from additional MCF-7 cell lines.

**Additional file 2:** Supplemental Table 1 showing results of Functional Enrichment Analysis of NFκB gene signatures in 4OHT-treated MCF-7 cell populations.

**Additional file 3:** Supplemental Table 2 showing results of Functional Enrichment Analysis of NFκB gene signatures in integrated parental and 4OHT-treated MCF-7 cell populations.

**Additional file 4:** Supplemental Table 3 showing DEGs from the NFκB+ cell population (i.e. Cluster 4).

**Additional file 5:** Supplemental Table 4 showing results of Functional Enrichment Analysis of Hallmark Signatures from MSigDB for the NFκB+ cell population (i.e. Cluster 4).

**Additional file 6:** Supplemental Table 5 showing correlation coefficients and p values of stress pathways vs. Hallmark NFκB signature in Cluster 4.

**Additional file 7:** Supplemental Table 6 showing results of Functional Enrichment Analysis of NFκB gene signatures in integrated MCF-7 cell populations from different laboratories.

**Additional file 8:** Supplemental Table 7 showing results of Functional Enrichment Analysis of NFκB gene signatures in integrated LTED (GSE122743) and 4OHT-treated MCF-7 cell populations.

## Data Availability

The datasets generated and analyzed in the current study are available in the Gene Expression Omnibus repository (GSE 181,812).

## Abbreviations

4OHT: 4-Hydroxytamoxifen
E2: Estradiol
ED: Estrogen deprivation
eIF2α: Eukaryotic translation initiation factor 2A
ER: Estrogen receptor
ET: Endocrine therapy
FEA: Functional enrichment analysis
ICI: ICI 182,780
ISR: Integrated stress response
NFκB: Nuclear factor kappa B
PDxO: Patient-derived xenograft organoid
peIF2α: Phosphorylated eukaryotic translation initiation factor 2A
scRNA-seq: Single-cell RNA sequencing
TAM: Tamoxifen
