# Supplementary material for: Selective pressure of endocrine therapy activates the integrated stress response through NFκB signaling in a subpopulation of ER positive breast cancer cells
Source: Breast Cancer Res. 2022 Mar 9;24:19. doi: 10.1186/s13058-022-01515-1 (PMC8908626; doi:10.1186/s13058-022-01515-1)
Supplement: Supplementary file 1 — Additional file 1: Supplemental Figure 1 showing integration of scRNA-seq datasets from additional MCF-7 cell lines. [file 13058_2022_1515_MOESM1_ESM.pdf]

Supplemental Figure 1. Integration of scRNA-seq datasets from additional MCF-7 cell lines. **(A)** Single-cell transcriptomes from 2911 untreated MCF-7 cells from the Frasor lab and 648 4OHT-treated MCF-7 cells were integrated and represented bi-dimensionally using Seurat package v.3.1. **(B)** The percent change in abundance of cell populations with 4OHT treatment relative to the total population for each group are shown. **(C)** FEA was performed for NFκB gene signatures with one representative example shown in box plots. AUC and P-values for other NFκB signatures are presented in Supplemental Table 6. **(D)** A custom gene signature was derived from differentially expressed genes in tumors of patients receiving neoadjuvant tamoxifen. The signature was used for FEA and the signature score per cluster is presented in boxplots. **(E-H)** The same analysis was performed for single cell transcriptomes from 769 untreated MCF-7 cells (GSE144320). **(I-L)** The same analysis was performed for single-cell transcriptomes from 1677 LTED MCF-7 cells (GSE122743). *P<0.05, **P<0.01, ***P<0.001, ****P<0.0001, ns=not significant.

S. Fig. 1

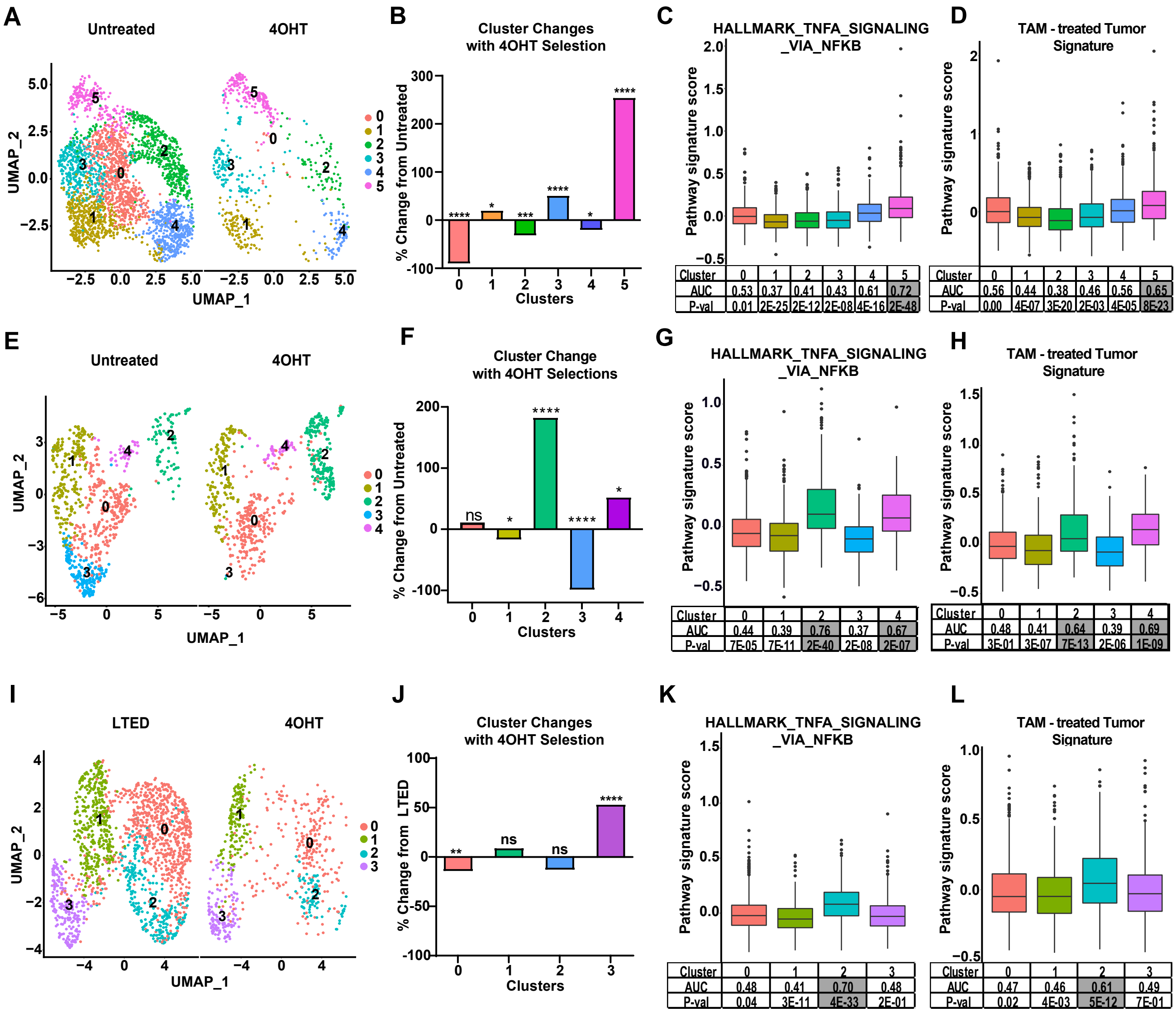
A
Untreated
4OHT
UMAP_2
UMAP_1
B
Cluster Changes with 4OHT Selestion
% Change from Untreated
Clusters
C
HALLMARK_TNFA_SIGNALING _VIA_NFKB
Pathway signature score
Cluster 0 1 2 3 4 5
AUC 0.53 0.37 0.41 0.43 0.61 0.72
P-val 0.01 2E-25 2E-12 2E-08 4E-16 2E-48
D
TAM - treated Tumor Signature
Pathway signature score
Cluster 0 1 2 3 4 5
AUC 0.56 0.44 0.38 0.46 0.56 0.65
P-val 0.00 4E-07 3E-20 2E-03 4E-05 8E-23
E
Untreated
4OHT
UMAP_2
UMAP_1
F
Cluster Change with 4OHT Selections
% Change from Untreated
Clusters
G
HALLMARK_TNFA_SIGNALING _VIA_NFKB
Pathway signature score
Cluster 0 1 2 3 4
AUC 0.44 0.39 0.76 0.37 0.67
P-val 7E-05 7E-11 2E-40 2E-08 2E-07
H
TAM - treated Tumor Signature
Pathway signature score
Cluster 0 1 2 3 4
AUC 0.48 0.41 0.64 0.39 0.69
P-val 3E-01 3E-07 7E-13 2E-06 1E-09
I
LTED
4OHT
UMAP_2
UMAP_1
J
Cluster Changes with 4OHT Selestion
% Change from LTED
Clusters
K
HALLMARK_TNFA_SIGNALING _VIA_NFKB
Pathway signature score
Cluster 0 1 2 3
AUC 0.48 0.41 0.70 0.48
P-val 0.04 3E-11 4E-33 2E-01
L
TAM - treated Tumor Signature
Pathway signature score
Cluster 0 1 2 3
AUC 0.47 0.46 0.61 0.49
P-val 0.02 4E-03 5E-12 7E-01
