# Supplementary material for: Selective pressure of endocrine therapy activates the integrated stress response through NFκB signaling in a subpopulation of ER positive breast cancer cells
Source: Breast Cancer Res. 2022 Mar 9;24:19. doi: 10.1186/s13058-022-01515-1 (PMC8908626; doi:10.1186/s13058-022-01515-1)
Supplement: Supplementary file 2 — Additional file 2: Supplemental Table 1 showing results of Functional Enrichment Analysis of NFκB gene signatures in 4OHT-treated MCF-7 cell populations. [file 13058_2022_1515_MOESM2_ESM.pdf]

Supplemental Table 1. FEA of NFkB gene signatures in 4OHT-treated MCF-7 cell populations.

Signatures	Populations			
	NFkB-		NFkB+	
	AUC	p-val	AUC	p-val
HALLMARK_TNFA_SIGNALING_VIA_NFKB	0.000192	1.08E-106	0.999808	1.08E-106
SANA_TNF_SIGNALING_UP	0.26553	7.78E-25	0.73447	7.78E-25
TIAN_TNF_SIGNALING_VIA_NFKB	0.297801	7.48E-19	0.702199	7.48E-19
PID_TNF_PATHWAY	0.359476	6.75E-10	0.640524	6.75E-10
BIOCARTA_NFKB_PATHWAY	0.387963	9.31E-07	0.612037	9.31E-07
REACTOME_TNF_SIGNALING	0.393676	2.91E-06	0.606324	2.91E-06
RUAN_RESPONSE_TO_TNF_UP	0.420445	0.000480276	0.579555	0.000480276
OSAWA_TNF_TARGETS	0.421477	0.000561865	0.578523	0.000561865
ZHOU_TNF_SIGNALING_30MIN	0.425779	0.001160607	0.574221	0.001160607
WANG_NFKB_TARGETS	0.429279	0.001824907	0.570721	0.001824907
WANG_TNF_TARGETS	0.429918	0.002163814	0.570082	0.002163814
BIOCARTA_RELA_PATHWAY	0.435689	0.004165386	0.564311	0.004165386
ZHOU_TNF_SIGNALING_4HR	0.442896	0.011353447	0.557104	0.011353447
