# Supplementary material for: Selective pressure of endocrine therapy activates the integrated stress response through NFκB signaling in a subpopulation of ER positive breast cancer cells
Source: Breast Cancer Res. 2022 Mar 9;24:19. doi: 10.1186/s13058-022-01515-1 (PMC8908626; doi:10.1186/s13058-022-01515-1)
Supplement: Supplementary file 3 — Additional file 3: Supplemental Table 2 showing results of Functional Enrichment Analysis of NFκB gene signatures in integrated parental and 4OHT-treated MCF-7 cell populations. [file 13058_2022_1515_MOESM3_ESM.pdf]

Supplemental Table 2. FEA of NFkB gene signatures in integrated parental and 4OHT-treated MCF-7 cell populations.

Signatures	Clusters									
	0		1		2		3		4	
	AUC	p-val	AUC	p-val	AUC	p-val	AUC	p-val	AUC	p-val
HALLMARK_TNFA_SIGNALING_VIA_NFKB	0.35121	3.35E-48	0.62245	9.18E-28	0.57029	1.52E-06	0.44577	0.00053	0.86652	1.30E-29
PID_TNF_PATHWAY	0.40463	1.06E-20	0.5618	4.32E-08	0.54112	0.00482	0.51665	0.28181	0.67436	8.50E-08
OSAWA_TNF_TARGETS	0.4666	0.00117	0.53325	0.00358	0.56688	4.04E-06	0.39696	4.52E-11	0.67194	1.12E-07
ZHOU_TNF_SIGNALING_4HR	0.48848	0.26432	0.42603	3.32E-11	0.4466	0.00025	0.69428	2.41E-35	0.66967	1.70E-07
TIAN_TNF_SIGNALING_VIA_NFKB	0.46805	0.00173	0.47921	0.064	0.57744	1.14E-07	0.49618	0.78327	0.6351	3.07E-05
REACTOME_TNF_SIGNALING	0.41065	2.06E-18	0.51066	0.34039	0.4662	0.01972	0.6996	5.03E-37	0.62802	7.00E-05
WANG_TNF_TARGETS	0.43421	9.92E-11	0.57302	6.86E-11	0.49506	0.74063	0.48933	0.49832	0.62321	0.00014
ZHOU_TNF_SIGNALING_30MIN	0.45711	2.64E-05	0.45862	0.00019	0.63642	1.02E-20	0.49776	0.89657	0.6227	0.00014
WANG_NFKB_TARGETS	0.44645	1.69E-07	0.60152	1.60E-19	0.4841	0.27148	0.43757	7.52E-05	0.53461	0.28244
BIOCARTA_NFKB_PATHWAY	0.46136	0.00016	0.56397	1.04E-08	0.49627	0.81187	0.47386	0.08993	0.48396	0.60049
BIOCARTA_RELA_PATHWAY	0.49548	0.66152	0.51783	0.11049	0.48704	0.3595	0.49471	0.74812	0.48205	0.58957
SANA_TNF_SIGNALING_UP	0.45607	1.90E-05	0.50842	0.46811	0.51837	0.21682	0.57704	8.55E-07	0.45447	0.15779
RUAN_RESPONSE_TO_TNF_UP	0.42957	5.04E-12	0.56711	2.08E-09	0.47289	0.0648	0.58294	1.20E-07	0.42762	0.02419
