# Supplementary material for: Selective pressure of endocrine therapy activates the integrated stress response through NFκB signaling in a subpopulation of ER positive breast cancer cells
Source: Breast Cancer Res. 2022 Mar 9;24:19. doi: 10.1186/s13058-022-01515-1 (PMC8908626; doi:10.1186/s13058-022-01515-1)
Supplement: Supplementary file 4 — Additional file 4: Supplemental Table 3 showing DEGs from the NFκB+ cell population (i.e. Cluster 4). [file 13058_2022_1515_MOESM4_ESM.pdf]

Supplemental Table 3. DEGs from NFkB+ cell population (i.e. Cluster 4).

gene	p_val	avg_logFC	pct.1	pct.2	p_val_adj	cluster
DDIT3	4.77E-40	136.6327	0.741	0.2	1.24E-35	4
UBC	8.74E-10	70.95105	0.963	0.862	2.28E-05	4
CEBPB	8.65E-13	63.67173	0.901	0.721	2.25E-08	4
SQSTM1	3.93E-09	43.28608	0.877	0.796	0.000102	4
SAT1	6.61E-08	37.51141	0.753	0.559	0.001721	4
GADD45A	1.27E-42	35.67176	0.79	0.222	3.32E-38	4
SLC3A2	1.32E-19	32.67205	0.914	0.618	3.43E-15	4
CDKN1A	3.98E-16	30.67074	0.827	0.468	1.04E-11	4
EIF4A2	5.78E-10	30.65032	0.802	0.64	1.51E-05	4
EFNA1	7.35E-09	23.61816	0.469	0.244	0.000191	4
TERF2IP	1.86E-07	22.5217	0.716	0.536	0.004832	4
KLF4	6.40E-09	21.6748	0.481	0.239	0.000167	4
SNHG12	2.10E-22	17.89958	0.617	0.216	5.47E-18	4
CIART	1.39E-16	16.46896	0.309	0.07	3.62E-12	4
ERRFI1	1.75E-08	16.17856	0.58	0.346	0.000456	4
TAF1D	6.81E-10	15.96818	0.753	0.658	1.77E-05	4
C3orf14	2.83E-08	15.49373	0.704	0.842	0.000737	4
EPB41L4A-/	5.89E-08	13.74222	0.778	0.627	0.001533	4
PTP4A1	5.92E-08	13.66664	0.864	0.733	0.001543	4
PPP1R15A	3.68E-19	13.48238	0.506	0.166	9.59E-15	4
NEU1	1.56E-12	12.22415	0.654	0.321	4.07E-08	4
HERPUD1	4.44E-21	12.18939	0.667	0.27	1.16E-16	4
TRIM16	6.49E-16	11.88288	0.605	0.272	1.69E-11	4
ZNF622	1.42E-10	11.80602	0.568	0.267	3.70E-06	4
BTG2	5.79E-12	11.77493	0.296	0.085	1.51E-07	4
ATF3	9.66E-103	11.59748	0.407	0.018	2.52E-98	4
HSPA5	1.07E-10	11.31252	0.827	0.693	2.79E-06	4
COG3	1.05E-11	10.19105	0.457	0.173	2.73E-07	4
GARS	1.28E-09	10.11743	0.704	0.46	3.33E-05	4
TSC22D3	5.44E-10	9.23638	0.556	0.272	1.42E-05	4
TGIF1	4.11E-08	9.171906	0.593	0.349	0.001072	4
YPEL5	2.13E-11	8.694498	0.556	0.271	5.56E-07	4
TRIB3	4.68E-08	8.044975	0.63	0.428	0.001218	4
PNRC1	1.98E-27	7.459222	0.605	0.185	5.16E-23	4
PDRG1	5.16E-08	7.405356	0.63	0.42	0.001343	4
RAB9A	1.12E-08	7.035688	0.469	0.227	0.000293	4
RND3	2.16E-07	6.609181	0.469	0.248	0.00563	4
CLK1	7.04E-10	6.297403	0.42	0.173	1.83E-05	4
ZNF408	2.08E-15	6.257185	0.444	0.145	5.42E-11	4
GOT1	6.27E-09	5.504904	0.494	0.243	0.000163	4
SESN2	5.92E-21	5.451434	0.42	0.103	1.54E-16	4
GTF2B	6.35E-13	5.420768	0.333	0.094	1.65E-08	4
MDM2	8.08E-09	4.574201	0.667	0.404	0.000211	4
HBP1	8.34E-08	4.302618	0.37	0.17	0.002172	4
TRIM16L	8.01E-18	4.102155	0.58	0.23	2.09E-13	4

TIGAR	5.64E-11	3.941701	0.481	0.195	1.47E-06	4
DNAJB9	9.57E-10	3.739624	0.506	0.271	2.49E-05	4
DDIT4	5.61E-18	3.672133	0.778	0.394	1.46E-13	4
DEDD2	3.86E-08	3.364972	0.42	0.193	0.001005	4
CDK7	1.46E-09	3.317818	0.407	0.17	3.81E-05	4
LURAP1L	5.19E-08	3.137078	0.469	0.256	0.001352	4
STX3	4.91E-08	3.09039	0.481	0.246	0.00128	4
RP11-670E	6.61E-09	2.86121	0.321	0.116	0.000172	4
TSC22D1	1.56E-07	2.740553	0.654	0.412	0.004064	4
RELB	7.65E-11	2.678591	0.272	0.077	1.99E-06	4
HMOX1	4.19E-29	2.508892	0.321	0.047	1.09E-24	4
TSG101	2.78E-07	2.501265	0.58	0.354	0.007248	4
CEP95	1.50E-07	2.402718	0.543	0.282	0.003901	4
FIP1L1	2.64E-09	2.27199	0.469	0.229	6.88E-05	4
LARP6	1.49E-09	1.912205	0.383	0.149	3.87E-05	4
MAP1B	2.13E-09	1.848441	0.284	0.09	5.54E-05	4
SUGT1	9.05E-09	1.478894	0.642	0.366	0.000236	4
BEX2	2.53E-18	1.442089	0.444	0.126	6.60E-14	4
RRAGC	1.43E-09	1.317111	0.457	0.205	3.73E-05	4
DUSP11	2.00E-07	1.121014	0.395	0.187	0.005217	4
SUPV3L1	1.11E-09	1.11886	0.309	0.104	2.89E-05	4
ZMYM5	8.02E-08	1.091688	0.395	0.177	0.002089	4
RNF25	5.27E-08	1.084061	0.333	0.129	0.001372	4
TRIM13	8.52E-09	1.080322	0.358	0.138	0.000222	4
CD55	1.36E-13	1.072337	0.358	0.106	3.55E-09	4
RP1-313I6.	1.53E-07	0.925051	0.358	0.152	0.003987	4
TSPYL2	1.62E-08	0.923136	0.358	0.141	0.000422	4
SLC7A11	1.47E-09	0.889459	0.481	0.239	3.82E-05	4
VCPKMT	1.96E-09	0.88374	0.321	0.111	5.09E-05	4
NFKBIB	1.58E-07	0.793256	0.494	0.259	0.004126	4
SARNP	3.31E-07	0.757739	0.333	0.143	0.008615	4
CLGN	1.97E-28	0.684248	0.259	0.03	5.12E-24	4
ZMAT3	1.09E-07	0.670809	0.395	0.176	0.002841	4
THAP1	2.79E-07	0.625481	0.321	0.13	0.007275	4
GLRX3	2.54E-07	0.487452	0.765	0.525	0.006627	4
CREBRF	3.33E-11	0.487162	0.272	0.074	8.68E-07	4
TROAP	2.53E-09	0.444731	0.136	0.48	6.59E-05	4
KDM3A	5.35E-09	0.388058	0.272	0.087	0.000139	4
WHSC1	6.58E-08	0.379093	0.099	0.401	0.001714	4
KLHL28	2.62E-07	0.35785	0.272	0.099	0.006813	4
