# Supplementary material for: Selective pressure of endocrine therapy activates the integrated stress response through NFκB signaling in a subpopulation of ER positive breast cancer cells
Source: Breast Cancer Res. 2022 Mar 9;24:19. doi: 10.1186/s13058-022-01515-1 (PMC8908626; doi:10.1186/s13058-022-01515-1)
Supplement: Supplementary file 5 — Additional file 5: Supplemental Table 4 showing results of Functional Enrichment Analysis of Hallmark Signatures from MSigDB for the NFκB+ cell population (i.e. Cluster 4). [file 13058_2022_1515_MOESM5_ESM.pdf]

Supplemental Table 4. FEA of Hallmark Signatures from MSigDB.

Signatures_TTvsParental	Cluster	
	4	
	AUC	p-val
HALLMARK_P53_PATHWAY	0.872084	1.71E-30
HALLMARK_TNFA_SIGNALING_VIA_NFKB	0.866519	1.30E-29
HALLMARK_UNFOLDED_PROTEIN_RESPONSE	0.81765	9.54E-23
HALLMARK_HEME_METABOLISM	0.808669	1.78E-21
HALLMARK_APOPTOSIS	0.805745	3.92E-21
HALLMARK_PI3K_AKT_MTOR_SIGNALING	0.742212	8.86E-14
HALLMARK_HYPOXIA	0.734996	4.15E-13
HALLMARK_ADIPOGENESIS	0.722722	5.06E-12
HALLMARK_UV_RESPONSE_UP	0.714877	3.48E-11
HALLMARK_MYOGENESIS	0.694098	2.48E-09
HALLMARK_XENOBIOTIC_METABOLISM	0.690056	3.94E-09
HALLMARK_INFLAMMATORY_RESPONSE	0.684102	1.40E-08
HALLMARK_CHOLESTEROL_HOMEOSTASIS	0.670432	1.41E-07
HALLMARK_PROTEIN_SECRETION	0.664232	3.71E-07
HALLMARK_DNA_REPAIR	0.663711	4.20E-07
HALLMARK_MTORC1_SIGNALING	0.655871	1.59E-06
HALLMARK_COMPLEMENT	0.627767	7.52E-05
HALLMARK_IL6_JAK_STAT3_SIGNALING	0.626368	0.000111
HALLMARK_COAGULATION	0.617009	0.000277
HALLMARK_ESTROGEN_RESPONSE_EARLY	0.608778	0.000826
HALLMARK_ALLOGRAFT_REJECTION	0.602963	0.001429
HALLMARK_ESTROGEN_RESPONSE_LATE	0.590806	0.004919
HALLMARK_APICAL_JUNCTION	0.586375	0.007381
HALLMARK_ANGIOGENESIS	0.57739	0.017252
HALLMARK_UV_RESPONSE_DN	0.577004	0.015779
HALLMARK_TGF_BETA_SIGNALING	0.566376	0.036071
HALLMARK_KRAS_SIGNALING_DN	0.566256	0.040498
HALLMARK_EPITHELIAL_MESENCHYMAL_TRANSITION	0.561174	0.058783
HALLMARK_MYC_TARGETS_V2	0.555604	0.082816
HALLMARK_FATTY_ACID_METABOLISM	0.549148	0.127485
HALLMARK_GLYCOLYSIS	0.54726	0.14097
HALLMARK_KRAS_SIGNALING_UP	0.53762	0.245691
HALLMARK_IL2_STAT5_SIGNALING	0.536498	0.25894
HALLMARK_SPERMATOGENESIS	0.533203	0.306278
HALLMARK_INTERFERON_GAMMA_RESPONSE	0.530922	0.340217
HALLMARK_REACTIVE_OXYGEN_SPECIES_PATHWAY	0.52979	0.366006
HALLMARK_HEDGEHOG_SIGNALING	0.528027	0.378086
HALLMARK_OXIDATIVE_PHOSPHORYLATION	0.519786	0.544096
HALLMARK_WNT_BETA_CATENIN_SIGNALING	0.515607	0.618114
HALLMARK_APICAL_SURFACE	0.514613	0.650417
HALLMARK_MYC_TARGETS_V1	0.501176	0.961077
HALLMARK_NOTCH_SIGNALING	0.495226	0.901807
HALLMARK_BILE_ACID_METABOLISM	0.490796	0.774776

HALLMARK_INTERFERON_ALPHA_RESPONSE	0.479291	0.539039
HALLMARK_ANDROGEN_RESPONSE	0.477138	0.461455
HALLMARK_MITOTIC_SPINDLE	0.476367	0.462981
HALLMARK_PEROXISOME	0.468521	0.336304
HALLMARK_PANCREAS_BETA_CELLS	0.458131	0.195404
HALLMARK_E2F_TARGETS	0.399762	0.002052
HALLMARK_G2M_CHECKPOINT	0.316854	1.61E-08
