# Supplementary material for: Selective pressure of endocrine therapy activates the integrated stress response through NFκB signaling in a subpopulation of ER positive breast cancer cells
Source: Breast Cancer Res. 2022 Mar 9;24:19. doi: 10.1186/s13058-022-01515-1 (PMC8908626; doi:10.1186/s13058-022-01515-1)
Supplement: Supplementary file 6 — Additional file 6: Supplemental Table 5 showing correlation coefficients and p values of stress pathways vs. Hallmark NFκB signature in Cluster 4. [file 13058_2022_1515_MOESM6_ESM.pdf]

Supplemental Table 5. Correlation coefficients and p values of stress pathways vs. Hallmark NFkB signature in Cluster 4.

Signatures	correlation HALLMARK_TNFA_SIGNALING_VIA_NFKB Cluster 4	p.val HALLMARK_TNFA_SIGNALING_VIA_NFKB Cluster 4
HALLMARK_APOPTOSIS	0.835809902	2.85E-22
HALLMARK_HYPOXIA	0.819267029	9.01E-21
HALLMARK_P53_PATHWAY	0.765558503	8.56E-17
HALLMARK_ADIPOGENESIS	0.673124907	5.82E-12
HALLMARK_UV_RESPONSE_UP	0.641462854	1.10E-10
HALLMARK_UNFOLDED_PROTEIN_RESPONSE	0.639992213	1.26E-10
HALLMARK_PI3K_AKT_MTOR_SIGNALING	0.507826739	1.30E-06
HALLMARK_HEME_METABOLISM	0.355749788	0.001116846
