# Supplementary material for: Selective pressure of endocrine therapy activates the integrated stress response through NFκB signaling in a subpopulation of ER positive breast cancer cells
Source: Breast Cancer Res. 2022 Mar 9;24:19. doi: 10.1186/s13058-022-01515-1 (PMC8908626; doi:10.1186/s13058-022-01515-1)
Supplement: Supplementary file 7 — Additional file 7: Supplemental Table 6 showing results of Functional Enrichment Analysis of NFκB gene signatures in integrated MCF-7 cell populations from different laboratories. [file 13058_2022_1515_MOESM7_ESM.pdf]

Supplemental Table 6. FEA of NFkB gene signatures in integrated untreated and 4OHT-treated MCF-7 cell populations. (Dr. Frasor lab)

Signatures	Clusters											
	0		1		2		3		4		5	
	AUC	p-val	AUC	p-val	AUC	p-val	AUC	p-val	AUC	p-val	AUC	p-val
HALLMARK_TNFA_SIGNALING_VIA_NFKB	0.533	0.00548	0.3709	1.89E-25	0.4094	1.58E-12	0.4261	2.12E-08	0.6106	3.47E-16	0.718	1.56E-48
WANG_TNF_TARGETS	0.522	0.06674	0.4353	1.90E-07	0.3874	1.19E-18	0.5421	0.00146	0.4589	0.00229	0.7088	1.03E-44
SANA_TNF_SIGNALING_UP	0.487	0.29622	0.4952	0.68887	0.4467	2.63E-05	0.5325	0.01379	0.4379	4.68E-06	0.6335	3.06E-19
OSAWA_TNF_TARGETS	0.521	0.06918	0.549	6.84E-05	0.3941	9.15E-17	0.4823	0.17302	0.4839	0.2263	0.5822	3.34E-08
TIAN_TNF_SIGNALING_VIA_NFKB	0.546	9.97E-05	0.4371	3.71E-07	0.4398	2.82E-06	0.4751	0.05897	0.5418	0.0021	0.5811	5.18E-08
RUAN_RESPONSE_TO_TNF_UP	0.485	0.20765	0.4611	0.00167	0.5411	0.00122	0.5238	0.07027	0.4367	3.04E-06	0.5701	2.94E-06
BIOCARTA_NFKB_PATHWAY	0.558	9.90E-07	0.444	6.10E-06	0.4589	0.00121	0.4834	0.21029	0.5012	0.94718	0.5654	9.75E-06
REACTOME_TNF_SIGNALING	0.433	1.46E-08	0.6176	2.28E-21	0.358	8.71E-29	0.5987	8.56E-14	0.4532	0.00055	0.5612	4.14E-05
ZHOU_TNF_SIGNALING_30MIN	0.541	0.00062	0.489	0.37133	0.4717	0.02688	0.4709	0.02866	0.4893	0.43657	0.5405	0.00708
BIOCARTA_RELA_PATHWAY	0.56	4.30E-07	0.4584	0.00081	0.462	0.00303	0.4886	0.39529	0.4955	0.71985	0.5373	0.01223
WANG_NFKB_TARGETS	0.54	0.00066	0.5177	0.15865	0.4685	0.01329	0.5023	0.82952	0.4437	3.46E-05	0.5196	0.21313
ZHOU_TNF_SIGNALING_4HR	0.534	0.00461	0.539	0.00166	0.3836	7.55E-20	0.5662	4.87E-07	0.4746	0.06039	0.4959	0.77967
PID_TNF_PATHWAY	0.559	5.95E-07	0.466	0.00609	0.4525	0.00019	0.546	0.00046	0.4807	0.1567	0.4858	0.3255

Supplemental Table 6. FEA of NFkB gene signatures in integrated untreated and 4OHT-treated MCF-7 cell populations. (Dr. Oesterreich lab (GSE144320))

Signatures	Clusters									
	0		1		2		3		4	
	AUC	p-val	AUC	p-val	AUC	p-val	AUC	p-val	AUC	p-val
HALLMARK_TNFA_SIGNALING_VIA_NFKB	0.435924	6.57E-05	0.386283	7.47E-11	0.75508	1.89E-40	0.365538	1.54E-08	0.665595	1.81E-07
WANG_TNF_TARGETS	0.417064	2.12E-07	0.406916	8.50E-08	0.74189	1.05E-36	0.494765	0.831817	0.478556	0.505573
OSAWA_TNF_TARGETS	0.470018	0.06386	0.394602	1.42E-09	0.688795	5.13E-23	0.452314	0.04049	0.53155	0.306322
SANA_TNF_SIGNALING_UP	0.412512	4.45E-08	0.503855	0.801499	0.653011	1.71E-15	0.475185	0.29031	0.457032	0.173918
ZHOU_TNF_SIGNALING_30MIN	0.46793	0.043236	0.450109	0.004373	0.598954	2.41E-07	0.506222	0.800005	0.507627	0.77597
TIAN_TNF_SIGNALING_VIA_NFKB	0.449102	0.001434	0.499473	0.9906	0.58026	2.71E-05	0.495713	0.833384	0.489851	0.750265
ZHOU_TNF_SIGNALING_4HR	0.483078	0.30481	0.469875	0.083645	0.535362	0.067487	0.540945	0.082772	0.495427	0.860765
BIOCARTA_NFKB_PATHWAY	0.462453	0.018956	0.509512	0.587708	0.520546	0.288646	0.553903	0.021899	0.464177	0.252602
REACTOME_TNF_SIGNALING	0.573858	4.46E-06	0.436859	0.000315	0.517599	0.352297	0.407419	0.000103	0.533505	0.28453
BIOCARTA_RELA_PATHWAY	0.467163	0.042631	0.509686	0.585362	0.513637	0.494561	0.549804	0.037155	0.47128	0.378743
RUAN_RESPONSE_TO_TNF_UP	0.463632	0.021156	0.474007	0.141123	0.477063	0.235602	0.630836	3.57E-08	0.557119	0.06731
WANG_NFKB_TARGETS	0.478264	0.166519	0.501724	0.897122	0.454887	0.017387	0.542401	0.068672	0.626916	6.31E-05
PID_TNF_PATHWAY	0.554315	0.000762	0.54149	0.018084	0.347243	1.87E-15	0.503269	0.884318	0.562407	0.045756
