# Supplementary material for: Selective pressure of endocrine therapy activates the integrated stress response through NFκB signaling in a subpopulation of ER positive breast cancer cells
Source: Breast Cancer Res. 2022 Mar 9;24:19. doi: 10.1186/s13058-022-01515-1 (PMC8908626; doi:10.1186/s13058-022-01515-1)
Supplement: Supplementary file 8 — Additional file 8: Supplemental Table 7 showing results of Functional Enrichment Analysis of NFκB gene signatures in integrated LTED (GSE122743) and 4OHT-treated MCF-7 cell populations. [file 13058_2022_1515_MOESM8_ESM.pdf]

Supplemental Table 7. FEA of NFkB gene signatures in integrated LTED (GSE122743) and 4OHT-treated MCF-7 cell populations.

Signatures	Clusters							
	0		1		2		3	
	AUC	p-val	AUC	p-val	AUC	p-val	AUC	p-val
HALLMARK_TNFA_SIGNALING_VIA_NFKB	0.47522	0.03558	0.40668	2.88E-11	0.69585	3.54E-33	0.47583	0.15706
ZHOU_TNF_SIGNALING_4HR	0.37906	7.84E-24	0.47345	0.05673	0.66056	9.64E-23	0.60482	3.74E-10
REACTOME_TNF_SIGNALING	0.47622	0.05162	0.40428	7.40E-12	0.65699	9.99E-22	0.51827	0.27506
WANG_TNF_TARGETS	0.47705	0.05606	0.43486	3.30E-06	0.63946	1.28E-17	0.49127	0.59435
SANA_TNF_SIGNALING_UP	0.56669	2.68E-08	0.40068	1.45E-12	0.6054	1.18E-10	0.40131	3.63E-09
TIAN_TNF_SIGNALING_VIA_NFKB	0.44974	2.60E-05	0.47285	0.05566	0.59518	5.77E-09	0.53669	0.02862
BIOCARTA_NFKB_PATHWAY	0.46817	0.00831	0.46835	0.0251	0.58949	5.22E-08	0.51319	0.43489
OSAWA_TNF_TARGETS	0.539	0.00113	0.41956	9.84E-09	0.58423	2.80E-07	0.45057	0.00316
ZHOU_TNF_SIGNALING_30MIN	0.39208	3.00E-19	0.49963	0.97353	0.57464	4.79E-06	0.63239	3.51E-15
BIOCARTA_RELA_PATHWAY	0.47079	0.01405	0.48134	0.19925	0.57072	1.62E-05	0.50922	0.58788
PID_TNF_PATHWAY	0.53417	0.00423	0.44621	0.00011	0.55516	0.00065	0.45239	0.004
WANG_NFKB_TARGETS	0.47644	0.05183	0.52508	0.07682	0.50612	0.72092	0.50352	0.81829
RUAN_RESPONSE_TO_TNF_UP	0.5272	0.02445	0.47688	0.09354	0.48394	0.33677	0.49704	0.88269
